# Spleen Tyrosine Kinase Inhibitor TAK-659 Prevents Splenomegaly and Tumor Development in a Murine Model of Epstein-Barr Virus-Associated Lymphoma

**DOI:** 10.1128/mSphereDirect.00378-18

**Published:** 2018-08-22

**Authors:** Osman Cen, Karuppiah Kannan, Jessica Huck Sappal, Jie Yu, Mengkun Zhang, Muzaffer Arikan, Ali Ucur, Duran Ustek, Yeter Cen, Leo Gordon, Richard Longnecker

**Affiliations:** a Department of Medicine, Division of Hematology/Oncology, Northwestern University Feinberg School of Medicine, Chicago, Illinois, USA; b Department of Microbiology-Immunology, Northwestern University Feinberg School of Medicine, Chicago, Illinois, USA; c Robert H. Lurie Comprehensive Cancer Center, Northwestern University Feinberg School of Medicine, Chicago, Illinois, USA; d Takeda Pharmaceuticals International Co., Cambridge, Massachusetts, USA; e Istanbul University, Institute of Experimental Medicine, Istanbul, Turkey; University of Michigan—Ann Arbor; University of North Carolina at Chapel Hill; University of North Carolina at Chapel Hill

**Keywords:** Burkitt’s lymphoma, EBV-related cancer, LMP2A, SYK, TAK-659

## Abstract

Epstein-Barr virus (EBV) is associated with several B and epithelial cell cancers. EBV-encoded latent membrane protein 2A (LMP2A) contributes to cellular transformation by mimicking B cell receptor signaling. LMP2A/MYC double transgenic mice develop splenomegaly and B cell lymphoma much faster than MYC transgenic mice do. In this study, we explored the potential therapeutic efficacy of a novel spleen tyrosine kinase (SYK) and FLT3 inhibitor TAK-659 for development of a treatment option for EBV-associated malignancies. In our transgenic model, TAK-659 treatment totally abrogated splenomegaly and tumor development in LMP2A/MYC mice in both pretumor and tumor cell transfer experiments. TAK-659 treatment killed tumor cells, but not host cells within the spleen and tumors. Furthermore, TAK-659 treatment abrogated metastasis of tumor cells into bone marrow. Our data also show that TAK-659 inhibits SYK phosphorylation and induces apoptosis in LMP2A/MYC tumor cells at low nanomolar concentrations. Therefore, TAK-659 may provide an effective therapeutic option for treatment of LMP2A-positive EBV-associated malignancies and should be explored further in clinical trials.

**IMPORTANCE** The novel SYK and FLT3 inhibitor TAK-659 prevents the enlargement of spleen and tumor development in a mouse model of EBV-associated lymphoma by counteracting the activation of cellular kinase SYK through the viral LMP2A gene by inducing cell death in tumor cells but not in nontumor cells. These findings indicate that TAK-659 may be a very effective nontoxic therapeutic molecule especially for EBV-positive hematologic malignancies.

## Abstract (precis)

The novel SYK and FLT3 inhibitor TAK-659 prevents the enlargement of spleen and tumor development in a mouse model of EBV-associated lymphoma by counteracting the activation of cellular kinase SYK through the viral LMP2A gene by inducing cell death in tumor cells but not in nontumor cells. These findings indicate that TAK-659 may be a very effective nontoxic therapeutic molecule especially for EBV-positive hematologic malignancies.

## INTRODUCTION

Lifelong latent Epstein-Barr virus (EBV) infection is highly common worldwide, as more than 95% of the world population has been infected (1, 2). In various parts of the world, a considerable number of hematologic and nonhematologic malignancies, such as Hodgkin’s lymphoma (HL), Burkitt’s lymphoma (BL), and nasopharyngeal carcinoma (NPC), have been associated with latent EBV infection (3–5). In immunocompromised individuals, such as HIV-infected patients, or pharmacologically immunosuppressed individuals, such as solid organ transplant recipients, EBV may lead to serious lymphoproliferative or lymphoid malignancies (6, 7).

Quite often, EBV positivity is a prognostic indicator for poor patient survival (8, 9). Current strategies for treatment of patients with EBV-positive malignancies are not much different from the corresponding EBV-negative malignancies (8). There is one exception, in the context of immune dysfunction, in which either immunosuppression is discontinued, for example as in organ transplant patients or the immune function is restored, such as in HIV/AIDS patients. In the case of transplant patients, the discontinuation of immunosuppression may risk the organ rejection and graft-versus-host disease (GVHD) (8). Recent developments in the adoptive immunotherapy field with tumor-specific T cells are promising, but while this field is still investigational, the process is tedious and complicated, as it needs to be individualized to each patient (10–13). Furthermore, it is not always successful (12, 14). Tumor-cell-specific antibodies, such as rituximab, have been shown to be effective in treating EBV-positive B cell lymphomas, but resistance is a problem (15–17). For example, expression of EBV latent membrane protein 1 (LMP1) renders tumor cells resistant to rituximab because of activation of Akt by LMP1 (18). Therefore, new alternative and specific approaches are needed to treat EBV-associated malignancies.

The most desirable way to develop EBV-specific treatment is to target viral genes that provide essential functions in EBV-infected cells. This should result in increased specificity for cells that are harboring EBV and that are dependent on viral gene products. Only a few studies have shown that targeting cellular kinases upregulated in the EBV-positive cells might prove useful (18–20). The EBV-encoded LMP2A protein is a very attractive candidate for inhibition. Previous studies have shown that LMP2A promotes B cell survival by acting as a B cell receptor (BCR) mimic (21–26). More importantly, a number of inhibitors have already been tested and have been shown to be effective in blocking LMP2A-mediated proliferative effects (19, 20). LMP2A is expressed during latent EBV infection (27–32) and in most EBV-associated malignancies, including BL, HL, NPC, and lymphoproliferative diseases (32–37) that arise upon immune dysfunction. Therefore, therapeutic agents targeting LMP2A-modulated cellular signaling components may provide effective treatment options for EBV-associated malignancies and proliferative disorders.

LMP2A provides baseline activation and survival signals when expressed in primary B cells, similar to the signals generated through an antigen-stimulated BCR (38, 39). Through its proline-rich and immunoreceptor tyrosine-based activation motif (ITAM)-like motifs, LMP2A recruits and activates cellular signaling molecules such as Lyn and SYK kinases, leading to B cell survival and proliferation (40–42). In our previous studies, we targeted LMP2A-induced cellular signaling for the treatment of EBV-associated malignancies using dasatinib to inhibit Lyn kinase activation or rapamycin to block phosphatidylinositol 3 (PI3)-kinase signaling, successfully blocking LMP2A-mediated B cell lymphoma in our murine model of EBV-driven BL (19, 20).

Another LMP2A-modulated cellular kinase important in the BCR signaling pathway is the spleen tyrosine kinase (SYK) (41, 43–46). It is required for the development of the lymphatic system in the embryo (47). SYK has been shown to be essential for BCR and Fc receptor signaling (48–50). Along with Lyn, SYK has an essential role in transducing signaling by BCR (51–55). Deregulated SYK has been implicated in various malignancies, including lymphoma, leukemia, and breast cancer (25, 56–58). Few investigational SYK inhibitors have shown limited efficacy in various hematologic malignancies, including lymphoma and leukemia (59, 60). However, these earlier SYK inhibitors also had significant side effects (59, 60). A recently developed SYK inhibitor, TAK-659 (61–64), is in clinical trials (NCT02000934, NCT02323113, NCT02834247, and NCT02954406), and preliminary clinical data indicate that it is nontoxic and highly effective against a subset of lymphomas (63–66). In this study, we investigated the effects of TAK-659 in our murine model of LMP2A-driven B cell lymphoma and found that it completely inhibited LMP2A-induced splenomegaly and tumor development in this model.

## RESULTS

### Development of lymphoma cell lines from LMP2A/MYC and MYC transgenic mice.

To understand the role of LMP2A in modulating cellular signaling in the murine MYC-induced lymphoma model, we established three different cell lines from each of the autochthonous lymphoma tumors spontaneously developed in LMP2A/MYC and MYC (λ-MYC) transgenic mice. Cytogenetic analysis showed various chromosomal aberrations but not any consistent patterns. None of the three LMP2A/MYC cell lines but all of the MYC cell lines showed chromosomal duplications (see Table S1 in the supplemental material). All three of the LMP2A/MYC cell line karyotypes had 40 chromosomes reflective of the healthy (normal) mouse karyotype. Double-minute acentric chromosomal fragments were also commonly observed (Table S1). In addition, a few polyploidy cells were observed in only one of the three LMP2A/MYC cell lines. In contrast, all three MYC cell lines showed duplicated chromosomes, generally multiple chromosomes with up to 50 chromosomes in some cells (Table S1).

10.1128/mSphereDirect.00378-18.4 TABLE S1  Cytogenetic analysis of MYC and LMP2A/MYC cell lines. The healthy mouse karyotype 2x is 38+XY, totaling 40 chromosomes. Download TABLE S1, PDF file, 0.01 MB. Copyright © 2018 Cen et al. 2018 Cen et al. This content is distributed under the terms of the Creative Commons Attribution 4.0 International license.

### LMP2A/MYC lymphoma cell lines have increased baseline tyrosine phosphorylation.

**FIG 1:**
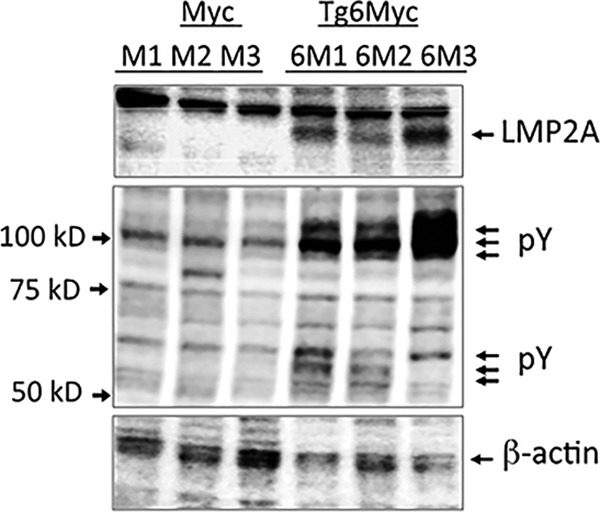
LMP2A/Myc cells have higher baseline tyrosine phosphorylation. Equal amounts of cell lysates from each of three different MYC cell lines (M1 to M3) and three different LMP2A/MYC cell lines (6M1 to 6M3) were electrophoresed and Western blotted with antibodies against LMP2A (top), total phosphotyrosine pY20 (pY; middle), or β-actin (bottom). The positions of molecular mass markers (in kilodaltons) are shown to the left of the blots.

The lysates from the MYC and LMP2A/MYC cell lines were Western blotted with pY20 antiphosphotyrosine antibody. The LMP2A/MYC cell lines showed several phosphotyrosine bands with increased intensity compared to the corresponding bands in the MYC cells (Fig. 1). We had previously shown that Lyn kinase was upregulated in LMP2A/MYC and that targeting Lyn provided a therapeutic option for this lymphoma (20). Given the importance of SYK in the generation and propagation of BCR-induced signaling and its modulation through LMP2A, we focused on SYK phosphorylation and its importance in the survival and tumorigenesis of LMP2A/MYC and MYC cells.

### A novel SYK inhibitor TAK-659 completely inhibits LMP2A-induced baseline SYK phosphorylation.

**FIG 2:**
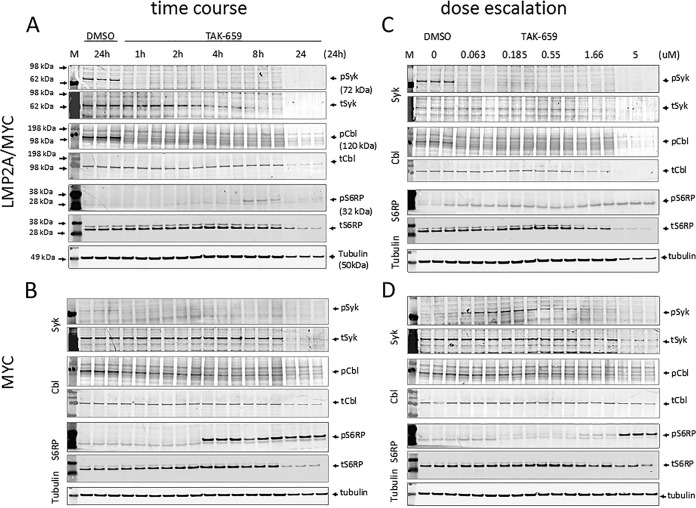
TAK-659 efficiently inhibits LMP2A-modulated cellular signaling. Cell lysates from pooled LMP2A/MYC (A and C) or MYC (B and D) cell lines cultured in triplicate in the absence (DMSO) or presence of 5 µM TAK-659 for different time points from 1 to 24 h (time course experiments [A and B]) or in the presence of different concentrations (0 to 5 µM) of TAK-659 for 24 h (dose escalation experiments [C and D]). The lysates from each culture were Western blotted with antibodies against the indicated kinase or protein. Kinase or protein abbreviations are as follows: pSYK, phosphorylated SYK (phosphorylated on pY525 or pY526); tSYK, total SYK; pCBL, phosphorylated CBL on Y774; tCBL, total CBL; pS6RP, phosphorylated S6RP on S235/S236; tS6RP, total S6RP. Lane M contains molecular weight markers. Please note that the nonspecific band with lower molecular weight on the pS6RP blot should not be confused with pS6RP.

To investigate SYK phosphorylation in the tumor cell lines, we used TAK-659, a small-molecule inhibitor of SYK kinase (62, 64). To test whether baseline SYK phosphorylation (phosphorylated SYK [pSYK], Y525/526) in LMP2A/MYC cells was inhibited by TAK-659, we cultured the cells with increasing concentrations of TAK-659 for various time points and assessed the levels of phosphorylated as well as total proteins for SYK and its downstream molecules c-CBL and ribosomal protein S6RP (S6 ribosomal protein) (Fig. 2; also see Fig. S1 in the supplemental material). Cells cultured in the absence of TAK-659 (dimethyl sulfoxide [DMSO]) showed two- to fourfold increased pSYK in LMP2A/MYC cells (Fig. 2A and C, topmost blots, and Fig. S1a) than in MYC cells (Fig. 2B and D, topmost blots, and Fig. S1a), while the levels of total SYK are very comparable except at the 24-h time point during which the levels of all total proteins decreased due to the high level of cell death (see below). TAK-659 at 5 µM totally inhibited this baseline pSYK in LMP2A/MYC cells within 1 h of the treatment (Fig. 2A, topmost blot, and Fig. S1a). The low-level baseline pSYK in the MYC cell lines was also similarly inhibited by 5 µM TAK-659 (Fig. 2B, topmost blot, and Fig. S1a). In a dose escalation experiment, concentrations as low as 63 nM TAK-659 were able to totally abrogate pSYK in LMP2A/MYC cells (Fig. 2C, topmost blot, and Fig. S1b), while pSYK in MYC cell lines was inhibited only at concentrations above 1 µM (1.66 µM) (Fig. 2D, topmost blot, and Fig. S1b). Interestingly, the lower concentrations of TAK-659 (60 to 200 nM) caused an increase in the pSYK level in the MYC cells (Fig. 2D, topmost blot, and Fig. S1b). Control blots with total SYK verified that this change in pSYK was not due to the difference in total protein levels, as the total levels of SYK and that of housekeeping protein tubulin were relatively stable (Fig. 2A to D, the total SYK [tSyk] blots and bottom blots for tubulin) except at the 24-h time point, where the levels of Syk was diminished likely due to the cells dying by apoptosis (see below). This decrease in total protein levels at the 24-h time point was apparent in Fig. 2 for all proteins tested, including tubulin albeit less pronounced. The ratio of densitometric value for each phosphorylated band to that of respective total protein was calculated using Image Studio Light software and graphed in Fig. S1 (Fig. S1a and b). These data indicate that the inhibition of SYK phosphorylation by TAK-659 is rapid and more sensitive in LMP2A/MYC cells than in MYC cells.

10.1128/mSphereDirect.00378-18.1 FIG S1  The intensity of each phosphorylated protein band in Fig. 2 was divided by the intensity of the corresponding total protein band and graphed. The graphs for pSYK (solid lines) and pCBL (dashed lines) in LMP2A/MYC (black) and MYC (gray) cells in time course (a) and dose escalation (b) experiments are shown. The graphs of pS6RP for LMP2A/MYC (black) and MYC (gray) cells in time course (c) and dose escalation (d) experiments are shown. Download FIG S1, PDF file, 0.1 MB. Copyright © 2018 Cen et al. 2018 Cen et al. This content is distributed under the terms of the Creative Commons Attribution 4.0 International license.

Due to high background in our Western blots and in particular the CBL blots, we ran positive controls to ensure that the bands analyzed were SYK and CBL. The pSYK, phosphorylated CBL (pCBL), tSYK, and total CBL (tCBL) bands correlated with the positive-control bands and ran at the appropriate molecular weights (MWs) (Fig. S2).

10.1128/mSphereDirect.00378-18.2 FIG S2  Verification of SYK and CBL bands in Western blots. Purified SYK (a) and CBL (b) protein or their 10-fold diluted mixtures (+) were loaded in lanes 7 and 6, respectively. Protein standards (molecular weight markers [M]; lane 1) were run on the same gel with various cellular lysates (lanes 2 to 4). To simplify the figure, the intervening nonrelated sample lanes were cropped (indicated by an asterisk). Lane 5 was intentionally not loaded with a sample to provide ample space with the controls. Download FIG S2, PDF file, 0.04 MB. Copyright © 2018 Cen et al. 2018 Cen et al. This content is distributed under the terms of the Creative Commons Attribution 4.0 International license.

### TAK-659 completely inhibits phosphorylation of CBL in LMP2A/MYC lymphoma cells.

LMP2A can also bind ubiquitin ligases, including the Nedd4 family ubiquitin ligases and the CBL (c-CBL) ubiquitin ligase (26, 67–70) via motifs found in the LMP2A amino-terminal domain. In our current study, we observed that the levels of phosphorylated CBL (pCBL) (Y774) in LMP2A/MYC cells showed a very similar pattern to that of pSYK. At baseline, CBL phosphorylation was higher in LMP2A/MYC cells (Fig. 2A) than in MYC cells (Fig. 2B), and TAK-659 inhibits pCBL much more readily in LMP2A/MYC cells than in MYC cells (Fig. 2A and B). The pattern of inhibition of pCBL mimicked that of pSYK, which was completely inhibited at 1 h (Fig. 2A) and also at very low concentrations of TAK-659 (0.06 µM [Fig. 2C]). The complete inhibition of pCBL in MYC cells in response to 5 µM TAK-659 was seen only after 8 h (Fig. 2B), while effective inhibition could be achieved at concentrations above 1.6 µM TAK-659 (Fig. 2D). The level of inhibition of pCBL in MYC cells was not reduced to the levels observed in the LMP2A/MYC cells (Fig. 2 and Fig. S1a and b). These data suggest that the presence of LMP2A in the signalosome containing SYK and CBL makes the complex more sensitive to the TAK-659 inhibition. The pCBL and tCBL bands correlated with the positive-control CBL band and with the protein ladder used (Fig. S2b).

### TAK-659 prevents phosphorylation of S6RP in LMP2A/MYC lymphoma cells.

We previously reported that inhibition of mTOR (mammalian target of rapamycin) with rapamycin decreased splenomegaly and tumor development in LMP2A/MYC tumors (19). S6 ribosomal protein (S6RP) is a regulatory component of the 40S ribosomal subunit that increases protein synthesis through its phosphorylation by p70S6K, an mTOR-regulated kinase (71–75). S6RP activity is increased by mitogenic signals but downregulated through cellular stress such as nutritional deprivation (71). It is involved in the regulation of various cellular processes. To test whether inhibition of proximal SYK activity with TAK-659 would lead to changes in downstream signaling, we assessed the phosphorylation of S6RP (pS6RP) (S235/236). Even though no baseline pS6RP was detected in either LMP2A/MYC or MYC lymphoma cells (Fig. 2A to D), S6RP phosphorylation was robustly observed with 5 µM TAK-659 treatment starting at 4 h only in the MYC lymphoma cells (Fig. 2B and Fig. S1c). pS6RP was not detected in LMP2A/MYC cells with early treatment time, and only a faint band was observed 8 h after TAK-659 exposure (Fig. 2A), indicating that TAK-659 can more readily counteract stress-induced S6RP activation in LMP2A/MYC cells, most likely through more complete inhibition of the mTOR pathway in the downstream axis of SYK. The induction of pS6RP in MYC cells was observed only with 5 µM TAK-659, as lower concentration of TAK-659 could not induce pS6RP either in MYC cells or in LMP2A/MYC cells (Fig. 2C and D and Fig. S1d).

### TAK-659 induces apoptosis more readily in LMP2A/MYC lymphoma cells.

**FIG 3:**
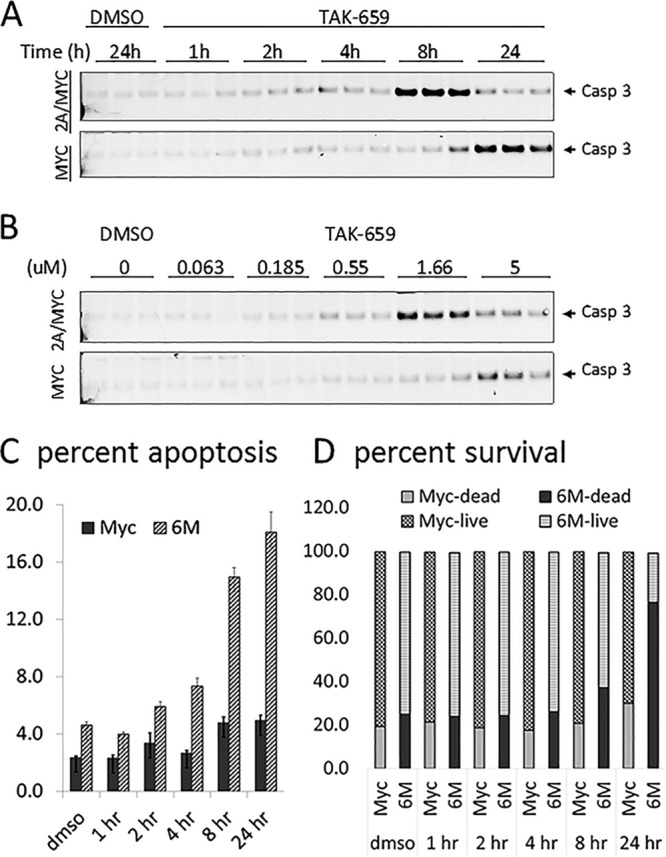
TAK-659 induces apoptosis in LMP2A/MYC cells. Cell lysates from time course (A) or dose escalation (B) experiments were processed as described in the legend to Fig. 2 and Western blotted with antibody for cleaved caspase 3 (Casp 3). In the same cultured cells as in panel A (time course experiment), the percent early apoptosis as YoPro1-positive cells within 7AAD-negative live gate (C) and the percentages of dead and live cell populations as 7AAD-positive and -negative cell population (D), respectively, as assessed by flow cytometry are shown (*n* = 3 for each data point). The data in Fig. 3C and D were graphed in Microsoft Excel.

To assess whether the inhibition of SYK phosphorylation has functional consequences, we analyzed the same protein lysates for cleaved caspase 3 (Casp3) as an indication of initiation of apoptosis (76, 77). At 5 µM concentration, TAK-659 induced Casp3 activation in the LMP2A/MYC cells which was readily apparent at 4 h and reached maximum levels at 8 h of treatment, while Casp3 activation in the MYC cells was not obvious until 24 h after the addition of the inhibitor (Fig. 3A and Fig. S3a). Similarly, Casp3 activation in LMP2A/MYC cells was observed at 0.5 µM and peaked at 1.6 µM concentration of TAK-659, while in MYC cells, Casp3 cleavage was observed only at the 5 µM concentration (Fig. 3B and Fig. S3b). To test the functional importance of caspase 3 cleavage, we also assessed apoptosis and cell survival in the same cultured cell samples by flow cytometry. In this data set, we analyzed live and dead cells and the early apoptotic cells within the live cell population. The data show that apoptosis was noticeable as early as 2 h of TAK-659 treatment and increased approximately fourfold by the 24-h time point in LMP2A/MYC cells. The level of apoptosis in MYC cells was noticeable only at 8- and 24-h time points and at lower levels. The apoptosis level in LMP2A/MYC cells was about three times that in observed in MYC cells (5 to 6% versus 15 to 18%) (Fig. 3C). The increased apoptosis correlated well with decreased percentage of live cells and increased percentage of dead cells. The decrease in the percentage of live cells in LMP2A/MYC cells was noticeable at 8 h and decreased from 75 to 80% to about 20% at 24 h (Fig. 3D). The percentage of live cells in MYC cells was also decreased but not to the extent in LMP2A/MYC cells (Fig. 3D). The apoptosis and cell death data also correlated well with the Western blotting data in Fig. 2, as at the 24-h time point, the levels of total SYK, CBL, and S6RP proteins (tSYK, tCBL, and tS6RP, respectively) decreased, and this decrease is more pronounced in LMP2A/MYC cells than in MYC cells. Even though the percent surviving cells in MYC cells is still relatively high at the 24-h time point, it is likely that some cell death occurred during the preparation/fixation/washing process for Western blotting, leading to additional protein loss.

10.1128/mSphereDirect.00378-18.3 FIG S3  Ratio of the intensity of cleaved caspase-3 (Casp3) band to the intensity of the corresponding tubulin band in LMP2A/MYC (black) and MYC (gray) cells in time course (a) and dose escalation (b) experiments. Download FIG S3, PDF file, 0.1 MB. Copyright © 2018 Cen et al. 2018 Cen et al. This content is distributed under the terms of the Creative Commons Attribution 4.0 International license.

### TAK-659 reverses LMP2A-induced splenomegaly and tumor development.

**FIG 4:**
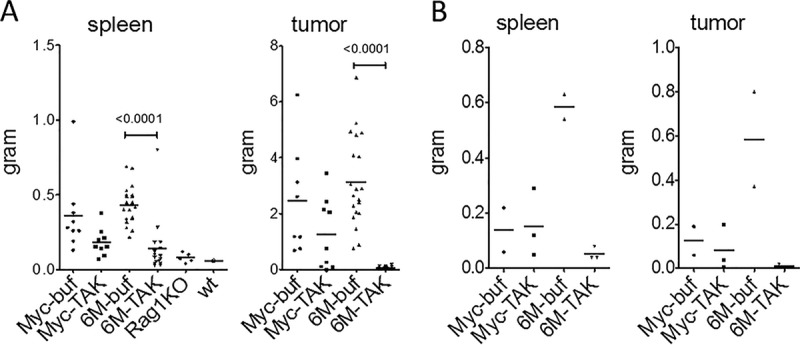
TAK-659 abrogates LMP2A-induced splenomegaly and tumor development in the Myc transgenic mouse model. The mass of spleen and tumor from the tumor cell transfer experiments (A) and from autochthonous experiments (B) were graphed for wild-type (wt) MYC (Myc) or LMP2A/MYC (6M) tumor-bearing mice treated with 0.5% methylcellulose (buffer [buf]) or TAK-659 (TAK). The masses of spleens and tumors were analyzed and graphed with GraphPad Prism. Each symbol represents the value for an individual mouse, and each bar shows the median for a group of mice. For panel A, *n* = 9 each for Myc-buf and Myc-TAK, *n* = 20 for 6M-buf, and *n* = 16 for 6M-TAK. For panel B, *n* = 2 for Myc-buf, *n* = 3 for Myc-TAK, *n* = 2 for 6M-buf, and *n* = 3 for 6M-TAK. (See also Table S2 in the supplemental material.)

Syngeneic transfer of LMP2A/MYC primary tumor cells into Rag1 knockout mice (Rag1KO mice) leads to tumor development and splenomegaly in the recipient Rag1KO mice within a few weeks. We have previously shown that the LMP2A-induced tumor development and splenomegaly are very sensitive to the Lyn inhibitor dasatinib and the mTOR inhibitor rapamycin (19, 20). To test whether SYK inhibition would also prevent splenomegaly and tumor development, we transferred LMP2A/MYC or MYC primary tumor cells into Rag1KO mice, and once the tumors were palpable, we treated the mice with either TAK-659 or methylcellulose buffer. Most interestingly and compatible with the strong SYK inhibition observed in the LMP2A/MYC cells, TAK-659 was able to completely inhibit splenomegaly and tumor development in the Rag1KO mice that had received LMP2A/MYC cells (Fig. 4A). The sizes of spleens in the TAK-659-treated mice were reduced threefold (from 434 mg to 143 mg), which resulted in spleen weights very similar to those that had not received tumor cells (Fig. 4A and Table S2). Similarly, but much more pronounced, the mass of tumors shrunk from 3,131 mg to 53 mg (Fig. 4A), which corresponds to an almost 60-fold decrease. The decreases in the masses of spleens and tumors in the mice that had received MYC primary lymphoma cells were also significant but not as dramatic as in the mice receiving LMP2A/MYC cells (Fig. 4A). In the mice with MYC tumors, the mean mass of the spleen was reduced from 361 mg to 182 mg and the mean mass of tumors was reduced from 2,468 mg to 1250 mg, respectively, both about twofold reduction (Table S2).

10.1128/mSphereDirect.00378-18.5 TABLE S2  Means and statistics of the sizes of spleens and tumors in the treated mice. Download TABLE S2, PDF file, 0.02 MB. Copyright © 2018 Cen et al. 2018 Cen et al. This content is distributed under the terms of the Creative Commons Attribution 4.0 International license.

LMP2A/MYC transgenic mice are born with enlarged spleens, and lymphadenopathy can be readily detected by 5 to 6 weeks of age, whereas MYC transgenic mice do not show splenomegaly or lymphadenopathy until they develop tumors. Tumor development in MYC mice is generally observed after 25 to 30 weeks of age with some not developing tumors even after 1 year of age (78, 79). As a proof of concept that TAK-659 inhibits LMP2A/MYC lymphoma development and to corroborate our tumor cell transfer data, we treated a limited number of MYC (16- to 24-week-old) and LMP2A/MYC (6- to 10-week-old) transgenic mice before they developed tumors. While the masses of spleens and inguinal lymph nodes in MYC mice without tumor were in the normal range and did not change with the TAK-659 treatment, the spleen mass of LMP2A/MYC mice was reduced from 585 mg in the buffer-treated group to 53 mg in the TAK-659-treated group, corresponding to an 11-fold reduction (Fig. 4B and Table S2). The spleen mass in treated mice was similar to the mass of the spleen of untreated, age-matched Rag1KO mice. Similarly, the size of lymph nodes in the MYC mice decreased from 125 mg to 83 mg with TAK-659 treatment, corresponding to a 1.5-fold decrease, while that of LMP2A/MYC mice decreased from 585 mg in the control group to 10 mg in TAK-659-treated mice (Fig. 4B, right panel), which again was similar to the lymph node size of untreated, age-matched Rag1KO mice. This corresponds to a 58-fold decrease in the lymph node size (Table S2). Together, these data indicate that TAK-659 specifically targets tumor and pretumor cells without obvious effects on nontumor cells.

### TAK-659 inhibits LMP2A-induced tumor cell survival *in vivo*.

**FIG 5:**
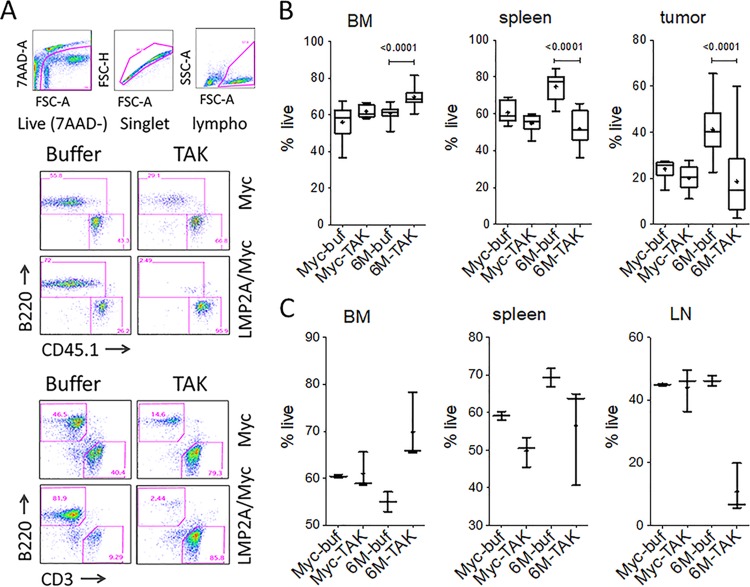
TAK-659 decreases cell survival in spleen and tumors but not in bone marrow. (A) A representative flow cytometric analysis scheme of single-cell suspensions from bone marrow (BM), spleen, and tumors/lymph nodes (LN) of mice in Fig. 4. The following sequential gating strategy was used to analyze the data presented in Fig. 5 and 6: live (7AAD negative), singlet, and lymphocyte/leukocyte (lympho). On the lymphocyte/leukocyte gate, the following populations were analyzed: B220 positive as tumor cells, CD45.1 positive (in tumor cell transfer model) as nontumor host cells (middle panel), or B220 positive as pretumor cells and CD3 (in autochthonous model) as nontumor host cells (bottom panel). (B) Percentages of live cells (7AAD negative) from tumor cell transfer groups from Fig. 4A are shown. (C) Percentages of live cells (7AAD negative) from autochthonous groups from Fig. 4B are shown. See “Data analysis and presentation” in Materials and Methods for explanation of the box plots. For panel B, *n* = 9 each for Myc-buf and Myc-TAK, *n* = 20 for 6M-buf, and *n* = 16 for 6M-TAK. For panel C, *n* = 2 for Myc-buf, *n* = 3 for Myc-TAK, *n* = 2 for 6M-buf, and *n* = 3 for 6M-TAK. (See also Table S2 in the supplemental material.)

To analyze the effects of TAK-659 on tumor and nontumor cells, we harvested bone marrow, spleens, and tumors from the same mice used in Fig. 4 and analyzed by flow cytometry. The tissues were processed into a single-cell suspension, labeled with various surface markers, and analyzed by flow cytometry using a sequential gating strategy as detailed in Materials and Methods and the legend to Fig. 5A. While there was no specific decrease in cell survival in bone marrow, the percentage of live cells dramatically decreased with the TAK-659 treatment in the spleens and tumors of LMP2A/MYC mice, in both tumor cell transfer (Fig. 5B) and autochthonous (Fig. 5C) models. We observed that in bone marrow, the percentage of live cells in the LMP2A/MYC mice increased with TAK-659 treatment, indicating that TAK-659 likely was not cytotoxic to bone marrow cells (Fig. 5B and C, BM panels).

### TAK-659 kills LMP2A-positive tumor cells but not nontumor cells *in vivo*.

**FIG 6:**
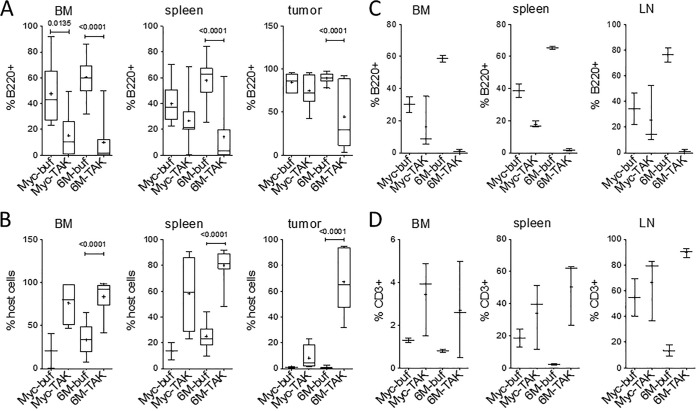
TAK-659 specifically targets tumor cells but not nontumor host cells. The percentages of tumor cells (B220 positive [B220+]) (A) and host cells (CD45.1 positive [CD45.1+]) (B) from the tumor cell transfer groups in Fig. 4A were analyzed. The percentage of pretumor cells (B220+) (C) and nontumor host cells (CD3+) (D) were analyzed in the autochthonous groups from Fig. 4B. For panel A, *n* = 5 each for Myc-buf and Myc-TAK, *n* = 7 for 6M-buf, and *n* = 6 for 6M-TAK. For panel B, *n* = 3 each for Myc-buf, *n* = 5 for Myc-TAK, *n* = 7 for 6M-buf, and *n* = 6 for 6M-TAK. For panels C and D, *n* = 2 for Myc-buf, *n* = 3 for Myc-TAK, *n* = 2 for 6M-buf, and *n* = 3 for 6M-TAK.

To assess what cell types might have been affected by TAK-659, we analyzed the tumor cells and nontumor host cells. In the tumor cell transfer model, the transferred tumor cells are B220 positive, CD19 positive, and CD45.2 positive but CD45.1 negative, while the host cells are B220 negative, CD19 negative, and CD45.2 negative but CD45.1 positive, as Rag1KO mice do not have any B or T cells. After sequential gating of the cells as described in Materials and Methods, we analyzed the percentages of B220-positive cells as tumor cells and CD45.1-positive cells as nontumor host cells. Initial comparison of B220, CD19, and CD45.2 showed that all three markers produced the same results (data not shown). We observed that in bone marrow, in both groups of mice that received MYC and LMP2A/MYC primary tumor cells, the percentage of B220-positive tumor cells was significantly decreased with TAK-659 treatment, but this decrease was much more pronounced in the mice with LMP2A/MYC cells (from 47% to 17% in MYC tumor cell recipients and from 60% to less than 10% in LMP2A/MYC tumor recipients) (Fig. 6A, BM graph). Correspondingly, the percentage of CD45.1-positive host cells was increased in the same group of mice (Fig. 6B, left panel). A similar pattern of cellular response was also observed in the spleens and tumors. In the spleens of mice with LMP2A/MYC tumor cells, the percentage of tumor cells was decreased from more than 60% in the buffer-treated mice to a few percent in the TAK-659 treated mice (Fig. 6A). In the same mice, the host cells increased from 25% to more than 80%, respectively (Fig. 6B). In the spleens of mice with MYC tumor cells, these numbers showed similar patterns, but they were less pronounced (Fig. 6A and B). In the tumors of mice with both MYC and LMP2A/MYC tumor cells that have been treated with buffer (control group), more than 90% of the cells were B220-positive tumor cells. With TAK-659 treatment, these numbers were decreased to less than 70% and 40%, respectively (Fig. 6A). In the same mice, the percentage of host cells were increased from a few percent in buffer-treated mice to about 10% in mice with MYC tumors and to more than 60% in mice with LMP2A/MYC cells that have been treated with TAK-659, respectively (Fig. 6B).

In the autochthonous model, a more pronounced effect was observed (Fig. 6C and D). In the bone marrow, spleens, and lymph nodes of the LMP2A/MYC transgenic mice, TAK-659 killed almost completely all B220-positive neoplastic cells (Fig. 6C) and correspondingly caused an increase in the nontumorigenic CD3-positive T cells (Fig. 6D). These data further support that TAK-659 is specifically targeting malignant and premalignant B cells but not healthy nonmalignant cells.

## DISCUSSION

EBV-associated malignancies pose considerable public health problems. They are less responsive to currently available treatment regimens compared to similar malignancies that are EBV negative (8, 80). Therefore, EBV-specific treatment options for EBV-positive malignancies are needed and may be particularly advantageous (8). We have focused on the role of EBV-encoded LMP2A in a murine model of Burkitt’s lymphoma. Using a Myc-based lymphoma model, we had previously shown that LMP2A accelerates Myc-induced lymphomagenesis (78, 79) and that targeting LMP2A-modulated cellular kinases in this model was efficacious (19, 20), encouraging our current studies exploring a new SYK inhibitor. In our current study, we showed an increase in SYK phosphorylation in LMP2A-positive MYC-induced lymphoma and that targeting SYK with a novel SYK inhibitor, TAK-659, completely inhibited splenomegaly and tumorigenesis. These results are unlike our previous work (19, 20) using rapamycin and dasatinib where treatment with these agents decreased splenomegaly and tumorigenesis but did not completely inhibit these phenotypes. The complete inhibition of the LMP2A-induced malignant phenotype with TAK-659 treatment demonstrated the highly significant therapeutic value of this agent. Our data strongly suggest that TAK-659 may prove to be an effective therapeutic option for EBV-associated LMP2A-positive malignancies.

Higher concentrations of TAK-659 were required for the inhibition of SYK phosphorylation, splenomegaly, and tumorigenesis in MYC-induced LMP2A-negative lymphoma, indicating that TAK-659 may also provide an important therapeutic option for other tumors that may not express LMP2A. This is in line with recent clinical studies using TAK-659 (65, 66) and indicates that TAK-659 may be particularly effective against malignancies with high baseline SYK activation.

It will be important to further characterize the mechanism by which TAK-659 prevents splenomegaly and tumorigenesis. A few interesting observations from our studies deserve more attention. First, the therapeutic effect of TAK-659 seems to be by inhibition of SYK phosphorylation and SYK activation of downstream signaling pathways. The activation of SYK by LMP2A made premalignant cells and tumor cells very sensitive to the effects of TAK-659, as 60 nM TAK-659 completely inhibited SYK phosphorylation in the LMP2A-positive cells while inducing SYK phosphorylation in the LMP2A-negative MYC lymphoma cells. In addition, TAK-659 induced apoptosis by upregulating caspase 3 cleavage (Fig. 3). Similarly, the presence of LMP2A made CBL phosphorylation more sensitive to inhibition by TAK-659. This effect mirrored that of pSYK, further supporting our earlier study showing the modulatory role of LMP2A in the signalosome involving SYK and CBL (68). It will be interesting to further analyze the effect of TAK-659 on the activation of the proteasomal degradation pathway through CBL, which is phosphorylated by activated SYK and in turn may lead to degradation of SYK and other cellular signaling molecules (81, 82).

Another interesting observation was the regulation of S6RP phosphorylation (pS6RP) by LMP2A and the response of this regulation to TAK-659. S6RP is normally phosphorylated in response to growth factors and mitogens by S6K downstream of the mTOR pathway (71, 74). The phosphorylation of S6RP, which is correlated with the increased protein synthesis and is upregulated in the cell cycle, is correlated with a poor prognosis in cancers (83, 84). At the baseline, LMP2A had little effect on S6RP. However, in the MYC lymphoma cells, there was an increased phosphorylation level of S6RP within 4 h of TAK-659 exposure. This might be a cellular response to the molecular stress exerted by TAK-659, probably through partial inhibition of the mTOR pathway. The absence of pS6RP after TAK-659 treatment in the LMP2A/MYC cells might be due to a more complete inhibition of the mTOR pathway, indicating that the presence of LMP2A might sensitize the cells to the therapeutic effect of TAK659. Delineating the mechanism of TAK-659 in inhibiting tumor development in our tumor model may reveal important information regarding the molecular mechanisms of TAK-659 action as well as the role of LMP2A in modulating cellular signaling. Results of these studies may provide a better understanding for developing treatment options for EBV-positive and EBV-negative hematologic malignancies.

### Conclusion.

Our data demonstrate the potential of TAK-659 in devising treatment strategies for EBV-positive and EBV-negative hematologic malignancies. It will be interesting to see the result of the currently open TAK-659 clinical trials for advanced solid tumors and lymphoma (NCT02000934) and for acute myelogenous leukemia (NCT02323113). Detailed stratification of the results on the basis of EBV and EBV-LMP2A may provide more valuable clinical data about the efficacy of TAK-659.

## MATERIALS AND METHODS

### Mice.

λ-MYC (MYC) and LMP2A/λ-MYC double transgenic mice (Tg6/MYC [6M]) have been previously described (38, 39, 78, 79, 85). λ-MYC mice were obtained from the National Cancer Institute (85). Rag1 knockout (Rag1KO) mice (B6.129S7-Rag1^tmMom^/J) (catalog no. 002216; Jackson Laboratory) were crossed with B6 CD45.1 mice (Bt.SJL-Ptprc^a^ Pepc^b^/BoyJ) (catalog no. 002014; Jackson Laboratory) through three rounds of backcrossing to obtain Rag1^−/−^ CD45.1^+/+^ mice. All mice were bred, housed, and used at the Northwestern University Center for Comparative Medicine in accordance and with approval of Institutional Animal Care and Use Committee guidelines. All *in vivo* studies and related protocols were approved by the Northwestern University’s Institutional Animal Care and Use Committee before the study was conducted.

### TAK-659.

TAK-659 was synthesized and characterized at Millennium/Takeda Oncology (62). For *in vitro* experiments, TAK-659 was dissolved in dimethyl sulfoxide (DMSO) at 100 mM (160 mg/ml), aliquoted, and stored at −20°C. For *in vivo* experiments, TAK-659 was suspended in sterile 0.5% methylcellulose at 125 mg/ml and kept at 4°C in aliquots. On each treatment day, aliquots were brought to room temperature, diluted further in 0.5% methylcellulose, and administered at 100 mg/kg of body weight to each mouse per day per oral gavage.

### Cell line development and culture.

Primary lymph node B cell tumor cells from MYC or LMP2A/MYC transgenic mice were continuously cultured in OPTI-MEM medium with 2% l-glutamine, 1% penicillin (Pen)-streptomycin (Strep), 25 µM beta-mercaptoethanol (BME), and 5 to 20% fetal bovine serum (FBS) in 12-well plates for various time points in a humidified 5% CO_2_ incubator at 37°C. The cells were washed one or two times a week and cultured with fresh medium each time. When growing foci were detected, the cells were diluted in fresh medium until they were dividing readily with more than 70% live cells in the culture; at this time, the cells were transferred to RPMI 1640 medium with 2% l-glutamine, 1% Pen-Strep, 25 µM BME, and 10% FBS in 25-cm^2^ flasks. Once they were growing well with >70% live cells (2 to 3 weeks), their frozen stocks were prepared. In most cases, tumor cells isolated from the transgenic mice were unable to be established as cell lines in culture. The overall success rate was about 20%. All cell lines are free of mycoplasma as tested by a mycoplasma test kit (LookOut mycoplasma PCR detection kit; Sigma-Aldrich, USA).

### Cytogenetic analysis of developed cell lines.

The LMP2A/MYC and MYC cell lines were individually cultured at 0.5 × 10^6^ cells/ml in RPMI 1640 medium in 25T flasks and incubated for 70 h as described above. At 70 h, to arrest cells at metaphase, colchicine was added to the cultures at 0.2 µg/ml and incubated for 2 more hours. The cells were then removed from the incubator and centrifuged. The pelleted cells were resuspended in 10 ml of 75 mM KCl for 15 min at 37C. The cells were pelleted at 400 × *g* for 10 min at room temperature and fixed with 1:3 acetic acid and methanol. The fixed cells were spread onto frosted microscope slides. Metaphase chromosomes were prepared according to the Giemsa-trypsin staining banding method (86). A light microscope equipped with an immersion oil objective of 100× magnification power and a digital camera was used to acquire digital images of selected metaphase chromosomes (Leica Microsystems, Germany). Karyotype analysis was performed on GTG banded metaphase chromosomes (87) (http://www.pathology.washington.edu/research/cytopages/idiograms/mouse/).

### Time course and dose escalation study of TAK-659.

To detect LMP2A-induced modulation in cell signaling, cells were pooled as follows. We initially grew three MYC cell lines and three LMP2A/Myc cell lines separately. Then, three million cells from each of the three MYC cell lines were pooled into one 10-mm culture plate and labeled Myc line. Three million cells from each of three LMP2A/MYC cell lines were similarly pooled and labeled LMP2A/Myc line. Each of these mixed cell lines were cultured in triplicate in RPMI 1640 medium as indicated above in 10-mm plates at 1 × 10^6^ cells/ml in the presence or absence of 5 µM TAK-659 for various time points from 1 h to 24 h (time course) or in the presence of various concentrations of TAK-659 from 0 to 5 µM (dose escalation) for 24 h. The cells were then pelleted and fixed in 0.5 ml of 4% paraformaldehyde for Western blotting analysis.

### PAGE and Western blotting.

The paraformaldehyde-fixed cultured cells were pelleted and lysed with radioimmunoprecipitation assay (RIPA) buffer containing protease and phosphatase inhibitors according to the standard protocol. Lysis supernatants were collected, and protein concentrations were determined by the bicinchoninic acid (BCA) method. Twenty micrograms of lysates for all phosphorylated proteins except 80 µg of lysates for pSYK and 10 µg for total proteins were run on SDS-polyacrylamide gels, transferred to polyvinylidene difluoride membranes optimized for fluorescence-based detection of Western blots (PVDF-FL membranes), and then blotted with antibodies for the indicated proteins or phosphoproteins. The blots were developed with Li-Cor Odyssey imaging system (Li-Cor Biosciences). The following antibodies were used in Western blotting: anti-total phosphotyrosine antibody pY20 (sc-503; Santa Cruz) at 1:1,000 dilution, pSYK (EP575-2, ab62350; Epitomic/Abcam, Inc.) at 1:500 dilution, total SYK (EP573Y, ab40781; Epitomic/Abcam) at 1:4,000 dilution, pCBL (Y774) (catalog no. 3555; Cell Signaling) at 1:1,000 dilution, total CBL (catalog no. 8447; Cell Signaling) at 1:1,000 dilution, pS6RP (S235/236) (catalog no. 8207; Cell Signaling) at 1:2,000 dilution, total S6RP (catalog no. 2217; Cell Signaling) at 1:2,000 dilution, cleaved caspase 3 (Casp3) (catalog no. 9664: Cell Signaling) at 1:1,000 dilution, and antitubulin (catalog no. 2148; Cell Signaling) at 1:2000 dilution. In all gels, SeeBlue Plus2 prestained protein standard (ThermoFisher) was used as molecular weight marker (M).

### Quantitation of Western blots.

The density of each band on each Western blot was analyzed using Image Studio Lite (Li-Cor). The densitometry value of each phosphorylated band for SYK, CBL, and S6RP was divided by the densitometry value of the corresponding total band to obtain a relative phosphorylation value for that band to find its relative phosphorylation level. The relative quantitation of caspase 3 was identified by dividing the densitometry value of each band by the densitometry value of the corresponding tubulin band. Within each marker, the intensities of the three replicates were averaged and graphed.

### Autochthonous transgenic model.

Our autochthonous model was used as previously published (19, 20, 78, 79). Briefly, nontumor transgenic λ-MYC (16- to 24-week-old) and pretumor Tg6/λ-MYC (6- to 10-week-old) mice were treated daily per oral gavage at 100 mg/kg of body weight/day (active TAK-659 ingredient) for 10 days. Control mice were treated with an equivalent volume (~200 μl) of 0.5% methylcellulose. The treatment is detailed below.

### Syngeneic tumor cell transfer model.

The syngeneic tumor cell transfer model was used as previously published (19, 20). Briefly, cervical or peripheral lymph node tumor cells from either λ-MYC or Tg6/λ-MYC mice were harvested, processed into single-cell suspensions, and frozen at −80°C. Cells were later thawed and washed, and 0.5 × 10^6^ cells in 200 µl of PBS were subcutaneously injected into the right flanks of recipient 12- to 18-week-old CD45.1 Rag-1KO mice under anesthesia. When the tumors became palpable, mice were assigned to control or experimental groups and treated and analyzed as detailed below.

### *In vivo* administration of TAK-659.

Mice were treated daily per oral gavage at 100 mg/kg/day (active TAK-659 ingredient) for 10 days. Briefly, each mouse in a treatment group received 100 mg/kg TAK-659 in 200 μl of 0.5% methylcellulose per oral gavage per day for 10 days. The mice in control groups received 200 μl of 0.5% methylcellulose alone per oral gavage per day for 10 days. One day after the last dose, the mice were sacrificed, and femur bones, tumors, and spleens were harvested, weighed, processed into single cells, and the cells were analyzed by multicolor flow cytometry.

### Flow cytometry analysis.

Single-cell suspensions were prepared from bone marrow, tumors, and spleens. Briefly, the ends of the bones were cut with a sterile pair of scissors and flushed with culture medium (RPMI 1640 medium plus 2% FBS [RPMI+2%FBS]) into 50-ml tubes. The spleen and tumors were smashed with a 10-ml syringe pistol and a 100-µm mesh filter into 50-ml tubes containing culture medium (RPMI+2%FBS). Then, red blood cells were lysed and washed three times with PBS. One million cells each from spleens, lymph nodes, or bone marrow were stained with the following antibodies: CD45.1 labeled with allophycocyanin (CD45.1-APC) (eBioscience), CD45.2 labeled with fluorescein isothiocyanate (CD45.2-FITC) (BD Biosciences), CD3-v500 (BD Biosciences), and B220-v450 (BD Biosciences) antibodies. The cells were treated with 7-aminoactinomycin D (7AAD) (Invitrogen) and analyzed in a FacsCANTO-II system (BD Biosciences). The data were analyzed with FlowJo. The following successive gating strategy was used in the given order for all samples: live cells (7AAD negative), singlet cells (forward scatter-pulse area [FSC-A] versus forward scatter-pulse height [FSC-H]), and lymphocyte/leukocyte (FSC-A versus side scatter-pulse area [SSC-A]). The percentages of CD45.1-, CD45.2-, B220-, and CD3-positive cells were determined within the lymphocyte/leukocyte gate. These sets of data were graphed using GraphPad Prism. For apoptosis, the cells were stained with 7AAD and YoPro1 (ThermoFisher) and analyzed in a FacsCANTO-II system. The 7AAD-positive cells were taken to be dead cells, and the 7AAD-negative cells were taken to be live cells, within which the YoPro1-positive population was considered early/pro-apoptotic cells. This last set of data was graphed using Microsoft (MS) Excel.

### Data analysis and presentation.

Unpaired Student’s *t* tests with two-tailed *P* values were used to analyze the data. Statistical analysis was performed using GraphPad Prism (version 5.0a; GraphPad Software Inc.). A *P* value of 0.05 or less was considered statistically significant. The data were graphed for each figure in the following format: the box plot for each group represents the interquartile range (25th to 75th percentiles), and the longer horizontal line in each box represents the median value. The mean is indicated with a plus sign and is a short horizontal line when it coincides with a vertical line or absent or a short vertical line when the median and mean correspond. The whiskers indicate minimum and maximum data points. When there are less than four data points in a group, a vertical line is shown instead of a box. Only *P* values of less than or equal to 0.05 are considered statistically significant and are shown as decimal numbers above the connected data points. The flow cytometry data on cell survival, cell death, and apoptosis from time course and dose escalation studies were graphed using MS Excel.

### Data availability.

The data sets generated during the current study are available from the corresponding author upon request.
