# Supplementary material for: Spleen Tyrosine Kinase Inhibitor TAK-659 Prevents Splenomegaly and Tumor Development in a Murine Model of Epstein-Barr Virus-Associated Lymphoma
Source: mSphere. 2018 Aug 22;3(4):e00378-18. doi: 10.1128/mSphereDirect.00378-18 (PMC6106053; doi:10.1128/mSphereDirect.00378-18)
Supplement: TABLE S1 [file sph004182623st1.pdf]

Table S1: Cytogenetic analysis of MYC and LMP2A/MYC cell lines: Normal mouse karyotype 2x is 38+XY, totaling in 40 chromosomes.

**Observations:** Like many other cell lines, various anomalies were noticed, prominent of which are listed below: The observations (and the number of cells they are observed).				
**LMP2A/MYC**			**MYC**	
**LM1**	40 chromosomes (1)<br>40+minute chromosomes (2)<br>Polyploidy (3)<br>Acentric fragments		**M1**	46 chromosomes (6)<br>45 chromosomes (8)<br>43 chromosomes (1)
**LM2**	40 chromosomes, many “double minute” acentric chromosomal pieces.<br>40+minute chromosomes (6)<br>Acentric fragments		**M2**	50 chromosomes (1)<br>47 chromosomes (1)<br>45 chromosomes (1)<br>44 chromosomes (3)<br>43 chromosomes (1)
**LM3**	40 chromosomes (5)<br>The least abnormalities		**M3**	Polyploidy (2)<br>46 chromosomes (1)<br>43 chromosomes (4)<br>42 chromosomes (4)<br>41 chromosomes (3)<br>41 chromosomes (1)<br>40 chromosomes (2)
