# Supplementary figures and images for: Spleen Tyrosine Kinase Inhibitor TAK-659 Prevents Splenomegaly and Tumor Development in a Murine Model of Epstein-Barr Virus-Associated Lymphoma
Source: mSphere. 2018 Aug 22;3(4):e00378-18. doi: 10.1128/mSphereDirect.00378-18 (PMC6106053; doi:10.1128/mSphereDirect.00378-18)

Figure S1

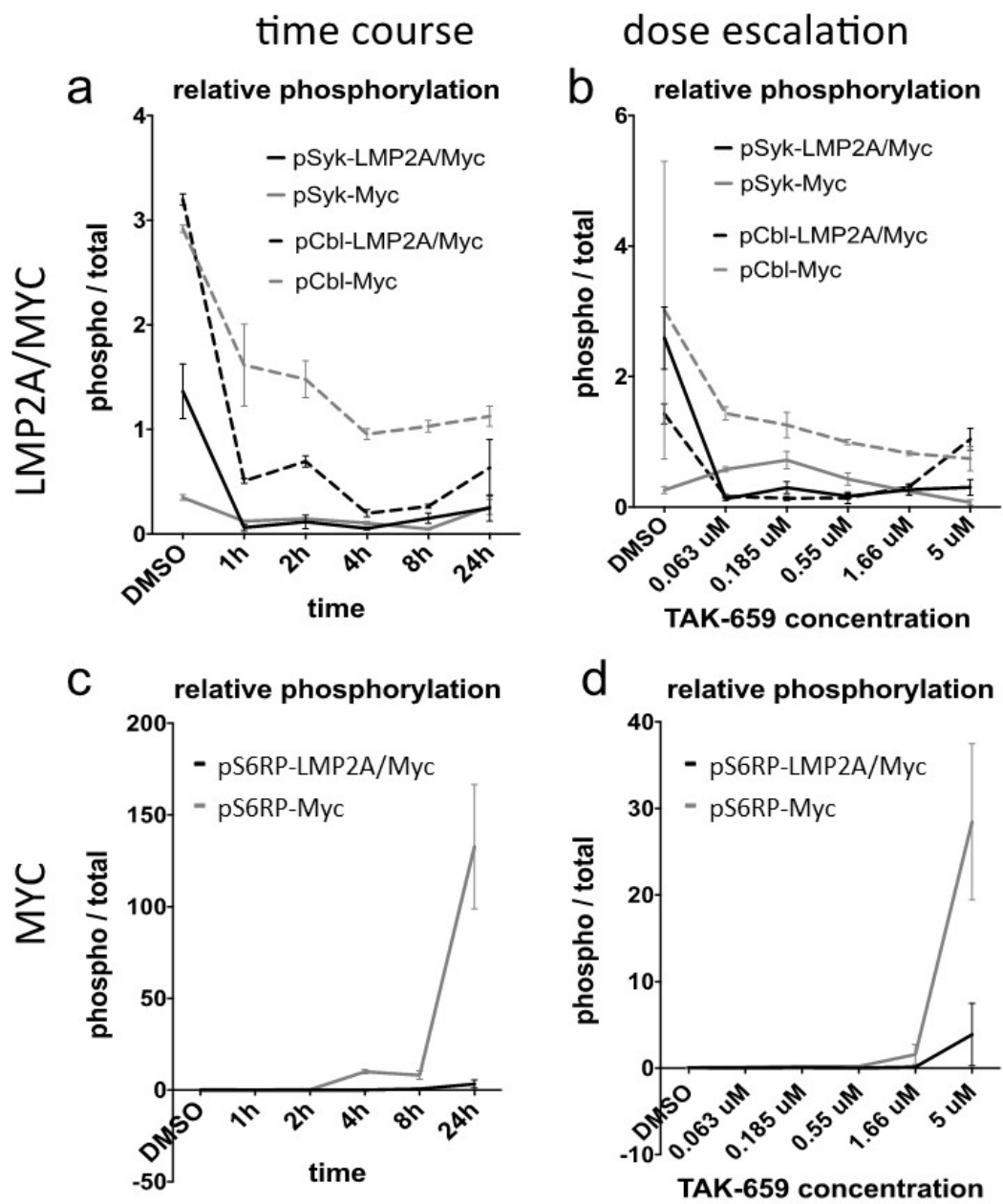

Supplement: FIG S1 [file sph004182623sf1.pdf]

**Figure S2**

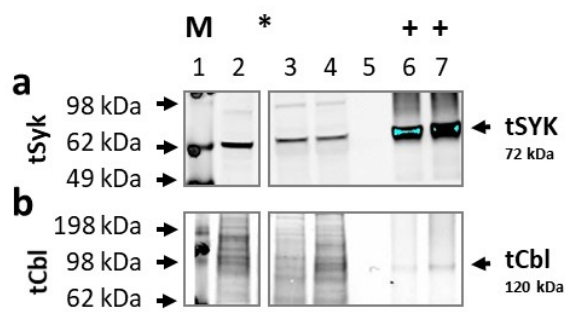

Supplement: FIG S2 [file sph004182623sf2.pdf]

**Figure S3**

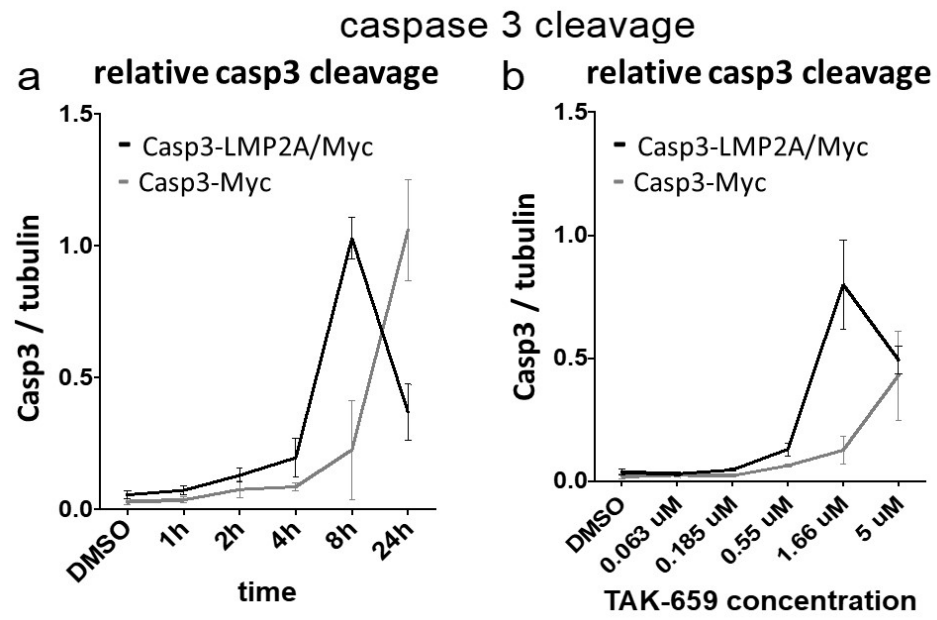

Supplement: FIG S3 [file sph004182623sf3.pdf]
