# Supplementary material for: Spleen Tyrosine Kinase Inhibitor TAK-659 Prevents Splenomegaly and Tumor Development in a Murine Model of Epstein-Barr Virus-Associated Lymphoma
Source: mSphere. 2018 Aug 22;3(4):e00378-18. doi: 10.1128/mSphereDirect.00378-18 (PMC6106053; doi:10.1128/mSphereDirect.00378-18)
Supplement: TABLE S2 [file sph004182623st2.pdf]

Table S2: The means and the statistics of the sizes of spleen and tumor in the treated mice

	Spleen mass						Tumor mass			
**TUMOR TRANSFER**	**n**	**mean**	**ST**	**p**	**fold decrease**		**mean**	**ST**	**p**	**fold decrease**
**6M-buffer**	20	0.434	0.028	<0.0001			3.131	0.359	<0.0001	
**6M-TAK**	16	0.143	0.047		3.03		0.053	0.014		59.07
**Myc-buffer**	9	0.361	0.084	0.064			2.468	0.688	0.144	
**Myc-TAK**	9	0.182	0.031		1.98		1.250	0.427		1.97
**PRE-TUMOR**	**n**	**mean**	**ST**	**p**			**mean**	**ST**	**p**	
**6M-buffer**	2	0.585	0.045				0.585	0.215		
**6M-TAK**	3	0.053	0.013				0.010	0.005		
**Myc-buffer**	2	0.140	0.080				0.125	0.065		
**Myc-TAK**	3	0.153	0.071				0.083	0.059		
